# Giant electrochemical actuation in a nanoporous silicon-polypyrrole hybrid material

**DOI:** 10.1126/sciadv.aba1483

**Published:** 2020-09-30

**Authors:** Manuel Brinker, Guido Dittrich, Claudia Richert, Pirmin Lakner, Tobias Krekeler, Thomas F. Keller, Norbert Huber, Patrick Huber

**Affiliations:** 1 Physics of Materials and High-Resolution X-Ray Analytics of the Structural Dynamics and Function of Matter, Hamburg University of Technology TUHH, 21073 Hamburg, Germany.; 2 Institute of Materials Research, Materials Mechanics, Helmholtz-Zentrum Geesthacht, 21502 Geesthacht, Germany.; 3 Deutsches Elektronen-Synchrotron DESY, 22607 Hamburg, Germany.; 4 Physics Department, University of Hamburg, 20355 Hamburg, Germany.; 5 Electron Microscopy Unit, Hamburg University of Technology, 21073 Hamburg, Germany.; 6 Center for Hybrid Nanostructures CHyN, University of Hamburg, 22607 Hamburg, Germany.

## Abstract

The absence of piezoelectricity in silicon makes direct electromechanical applications of this mainstream semiconductor impossible. Integrated electrical control of the silicon mechanics, however, would open up new perspectives for on-chip actuorics. Here, we combine wafer-scale nanoporosity in single-crystalline silicon with polymerization of an artificial muscle material inside pore space to synthesize a composite that shows macroscopic electrostrain in aqueous electrolyte. The voltage-strain coupling is three orders of magnitude larger than the best-performing ceramics in terms of piezoelectric actuation. We trace this huge electroactuation to the concerted action of 100 billions of nanopores per square centimeter cross section and to potential-dependent pressures of up to 150 atmospheres at the single-pore scale. The exceptionally small operation voltages (0.4 to 0.9 volts), along with the sustainable and biocompatible base materials, make this hybrid promising for bioactuator applications.

## Teaser

A silicon-polymer hybrid shows large, reversible electrostrain at remarkably small operation voltages in aqueous electrolyte.

## INTRODUCTION

An electrochemical change in the oxidation state of polypyrrole (PPy) can increase or decrease the number of delocalized charges in its polymer backbone (*1*). Immersed in an electrolyte, this is also accompanied by a reversible counter-ion uptake or expulsion and thus with a marcroscopic contraction or swelling under electrical potential control, making PPy one of the most used artificial muscle materials (*1*–*5*).

Here, we combine this actuator polymer with the three-dimensional (3D) scaffold structure of nanoporous silicon (*6*–*8*) to design, similarly as found in many multiscale biological composites in nature (*9*), a material with embedded electrochemical actuation that consists of a few light and abundant elemental constituents (i.e., H, C, N, O, Si, and Cl).

## RESULTS

**Fig. 1:**
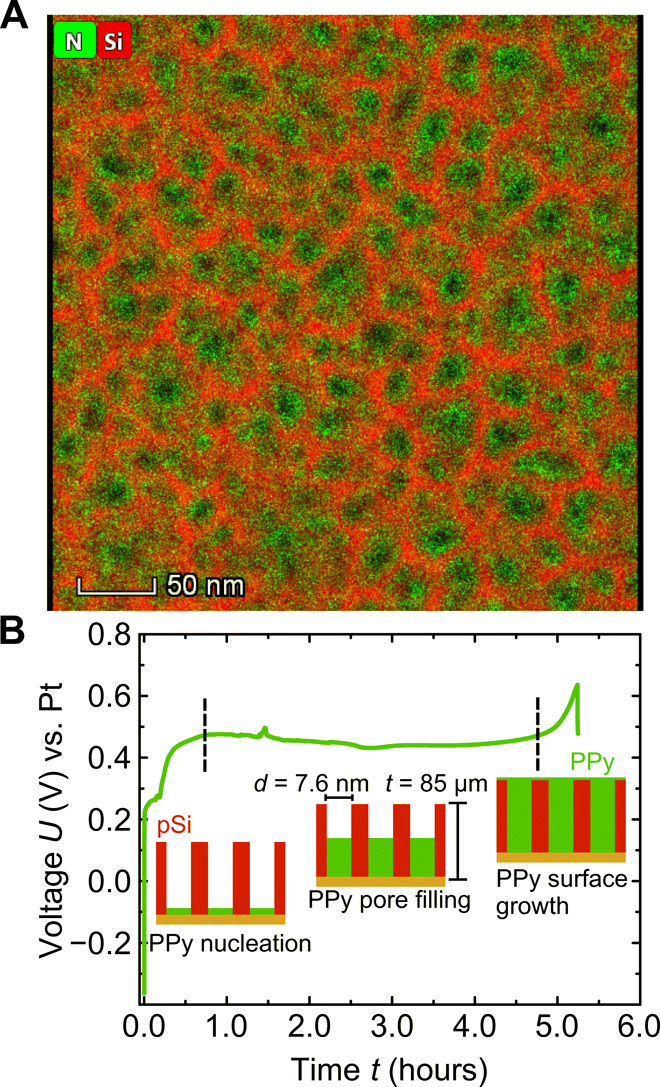
Synthesis of a nanoporous PPy-silicon material. (**A**) High-angle annular dark-field scanning TEM top view on a nanoporous silicon membrane filled by electropolymerization with pyrrole. The green and red color codes indicate the N and Si concentration resulting from EDX detection measurements, respectively. (**B**) Voltage-time recording during galvanostatic electropolymerization of pyrrole in nanoporous silicon, with mean pore diameter *d* and thickness *t*. Characteristic regimes are indicated and discussed in the main text.

In a first step, the porous silicon (pSi) membrane is prepared in an electrochemical etching process of doped silicon in hydrofluoric acid. The resulting pores are characteristically straight and perpendicular to the silicon surface. The analysis of a nitrogen sorption measurement yields a mean pore diameter of *d* = 7.2 nm and a porosity of Φ = 50%. This corresponds to 170 billion channels/cm^2^. Scanning electron microscopy profiles give a homogeneous sample thickness of *t* = 85 ± 1 μm. The pSi membrane is then filled with PPy by electropolymerization of pyrrole monomers. The graph in Fig. 1B shows the time-dependent voltage during the galvanostatic polymerization process. The curve exhibits characteristic transient regimes. Initially, the voltage shows an increase from its open circuit potential of around −0.4 V to approximately 0.5 V. This potential transition is attributed to the polymer nucleation at the bottom of the pore and a partial oxidation of the pSi. The ensuing constant voltage plateau is then related to a constant deposition of PPy inside the pores. The voltage increase at 5 hours is associated with the termination of pore filling and the onset of polymerization of pyrrole on the membrane surface. The geometrical constraint of the highly asymmetrical pores benefits a chain-like polymer growth and inhibits a branching of the polymer, which leads to a lower electrical resistance (*10*). Conversely, a polymer branching is more likely to appear during the unrestricted polymerization on the surface leading to a higher electrical resistance. Thus, a transition to a higher voltage indicates the polymerization of bulk PPy on the surface. To prevent this layer formation, the current is switched off when the voltage starts to increase toward a higher voltage, as it is the case at 5 hours. A transmission electron micrograph (TEM) with an overlayed energy-dispersive x-ray (EDX) spectroscopy signal of the resulting membrane material indicates a homogeneous PPy filling of the random pSi honeycomb structure (see Fig. 1A).

**Fig. 2:**
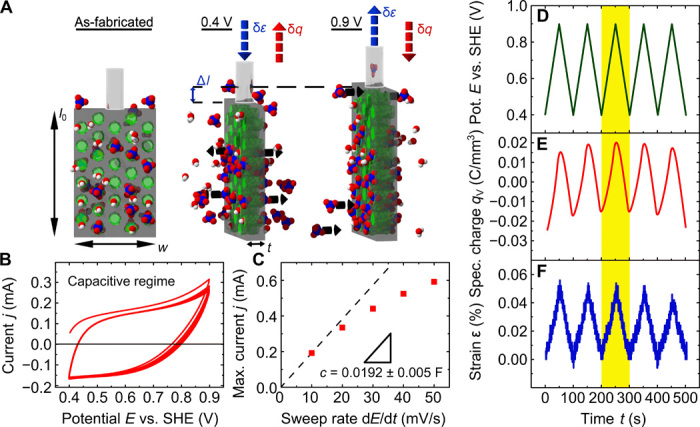
Electrochemical actuation experiments. (**A**) Schematics of the electroactuation experiments on the pSi membrane (gray) filled with PPy (green) immersed in an aqueous electrolyte [HClO_4_ (blue and red) and H_2_O (red and white) molecules]. The dimensions of the as-fabricated membrane, on the left, are length *l*_0_, width *w*, and thickness *t*. The middle part illustrates the case where a voltage of 0.4 V is applied and the ${\text{ClO}}_{4}^{-}$ anions are expelled from the PPy, resulting in the contraction of the sample. Vice versa, in the right part, a voltage of 0.9 V is applied, and the anions are incorporated, followed by the subsequent expansion of the sample. The change in length is indicated by Δ*l*. (**B**) The graph depicts an exemplary cyclic voltammetry of a pSi-PPy membrane in 1 M HClO_4_ electrolyte. The current *j* is plotted against the applied potential *E* measured versus the SHE. The potential sweep rate is 10 mV/s. (**C**) The graph depicts the mean values for the maximal current density of *j* plotted against varying potential sweep rates d*E*/d*t* from 10 to 50 mV/s. The dashed line indicates a linear regression of the data points, which yields the capacitance c* as the slope. Depicted on the right are (**D**) five representative potential cycles *E*, (**E**) the resulting volumetric charge *q*_V_, and (**F**) the introduced effective strain ε of the nanoporous membrane.

To characterize the actuorics of the resulting hybrid material, we perform dilatometry measurements in an in situ electrochemical setup. The sample is immersed in perchloric acid (HClO_4_; with a concentration of 1 M) and positioned, so that the pores are pointing in a horizontal direction (see Fig. 2A and fig. S1C). The quartz probe of the dilatometer is positioned on the top of the sample to measure the sample length *l*. The sample length *l*_0_ that is thus in contact with the perchloric acid is *l*_0_ = 0.626 ± 0.005 mm, with a width of the sample of *w* = 3.49 ± 0.01 mm. The measurements are performed with a potentiostat in a three-electrode setup. All potentials are indicated versus the standard hydrogen potential (SHE).

Before and during the actuoric dilatometry measurements, the electrochemical characteristics of the hybrid system are determined by recording cyclic voltammograms (CVs) in the potential range from 0.4 to 0.9 V with a sweep rate of 10 mV/s (see Fig. 2B). The ordinate shows the current *j*. The pSi-PPy membrane exhibits the characteristics of a capacitive charging of the PPy, with *j* quickly moving toward a constant value and the absence of true oxidation and reduction peaks (*11*). Higher voltages are not applied, since it leads to an overoxidation and partial destruction of PPy (*12*). Lower voltages could be applied, and a true reduction peak should be visible below 0 V. However, an aqueous electrolyte is used, which limits the CVs lower sweep potential to values above 0 V, since electrolysis of the water sets in below (*13*). However, Faradaic current contributions are present in the depicted CV since the currents toward the upper limit and the lower limit are not completely constant, as they ideally should be for pure capacitive behavior. The Faradaic currents can be attributed to an oxidative process that consumes charge. Likely, it is an oxidation of the silicon pore walls. We account for this effect by subtraction of the charge that is consumed in this way.

In Fig. 2B, we present the dependence of the maximal current *j*_max_ recorded in the CV, plotted against an increasing scan rate $\overset{\cdot }{E}=dE/dt$. The nonlinear course of the relation shows that a diffusion limitation, discussed in more detail below, affects the capacitive charging of the PPy. The limitation is larger for higher sweep rates. The capacitance *c* is defined as *c* = δ*q*/δ*E*, where *q* is the charge and *E* is the applied potential. The linear slope of *j*_max_ plotted against the sweep rate gives then *c* = Δ*j*_max_/Δ(d*E*/d*t*). Because of the diffusion limitation, the capacitance is determined by a linear regression of the first data point only, *c* = 0.0192 ± 0.005 F. Assuming a volume specific capacity of 0.24 F/mm^3^, as reported for PPy films doped with the same anion, the PPy confined in the pores has a volume of *V*_PPy_ = 0.08 mm^3^. This corresponds to a reasonable pore filling degree of 86.2%.

Recording the sample length change while recording the CVs allows as a detailed characterization of the electrochemical actuation (see Fig. 2, D to F). While *E* is reversibly changed from 0.4 to 0.9 V, the current and thus the transferred charge are recorded. The charge normalized to the sample volume *V*_sample_ is denoted as *q*_V_ and shown in Fig. 2E. The transferred charge coincides with *E*. So, increasing the potential leads to an increased insertion of anions into the PPy, and, vice versa, decreasing the potential releases anions from the PPy. The resulting length variation normalized to the sample length ε = Δ*l*/*l*_0_ = (*l* − *l*_0_)/*l*_0_ is shown in Fig. 2F. The repetitive change in sample length coincides linearly with *E* and *q*_V_. So, the incorporation of anions leads to an expansion of the sample, while the release of the anions lets the sample contract. The amplitude of the length change shows no signs of a decrease and is exhibiting an excellent reproducibility and thus a repeatable and robust mechanical actuation functionality. The sign of the strain-charge coupling is positive, meaning that sweeping the potential in positive direction leads to an expansion of the sample and vice versa.

**Fig. 3:**
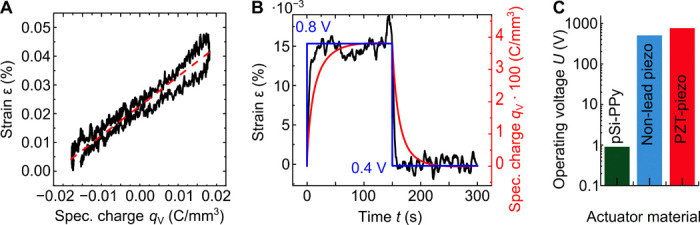
Static and dynamic electrochemical performance parameters. (**A**) Strain ε versus deposited volumetric charge *q*_V_. (**B**) Step coulombmetry to determine the electroactuation kinetics. The applied potential *E* is changed in a step-like fashion from 0.4 to 0.8 V and backward, while the thereby incorporated volumetric charge *q*_V_ and the caused strain ε are measured versus time. (**C**) Operating voltage *U* needed to achieve a strain amplitude of ε = 0.05% for the sample length of *l* = 0.626 mm for the pSi-PPy sample in comparison with high-performance piezoelectric lead-free (*22*) and lead-containing [lead zirconate titanate (PZT)] ceramics (*21*).

Additional actuation details can be inferred by averaging the volumetric charge density *q*_V_, normalized to the sample volume, and the strain ε over the five CV cycles, separately for positive- and negative-pointing sweeps. The peak-to-peak amplitude of the relative length variation ε over the five cycles is 0.05 ± 0.002%, which yields an absolute amplitude of 0.313 ± 0.0013 μm. Figure 3A provides a plot of ε versus *q*_V_, both recorded in the associated potential range of Fig. 2D. The relation between the two is highly linear as well in agreement with the robust actuation functionality inferred above. The corresponding linear coupling parameter relating the strain response to the incorporated anions by the volumetric charge (*14*) is A* = dε/d*q*_V_ = 0.01029 ± 0.00009 mm^3^/C.

To analyze the actuation kinetics, we perform step coulombmetry. The response to a square potential from 0.4 to 0.8 V is depicted in Fig. 3B. The associated strain ε saturates at 0.015%, while the volumetric charge *q*_V_ saturates at 0.04 C/mm^3^. When the potential is decreased to 0.4 V, ε decreases to 0%, and the volumetric charge reaches 0.0 C/mm^3^. Simple exponential functions can be used to fit the course of ε and *q*_V_ for both the increase and decrease, respectively. The time constants τ yield the speed of the respective processes, i.e., the charging and discharging of *q*_V_ and the respective reaction of ε: τ_*q*_V_, incr_ = 16.52 ± 0.07 s for the volumetric charge increase, τ_ε, incr_ = 4.26 ± 0.17 s for the strain increase, τ_*q*_V_, decr_ = 14.31 ± 0.07 s for the volumetric charge decrease, and τ_ε, decr_ = 2.8 ± 0.1 s for the strain decrease. The strain response is thus faster than the charging and discharging process by almost an order of magnitude. Two effects likely contribute to this observation. First, the PPy reaches, at least partially, its yield limit, and plastic deformation of the PPy sets in. Thus, the whole sample is not expanding further, although counter ions are still incorporated into the polymer, a hypothesis that we address in more detail in the micromechanical analysis below. Second, the already mentioned diffusion limitation hinders a faster transfer of the anions to the PPy. An estimation of the anion drift dynamics based on the self-diffusion coefficient *D* = 1.53 · 10^−9^ m^2^/s (*15*) of ${\text{ClO}}_{4}^{-}$ ions in PPy, as determined by molecular dynamics simulations (*16*), supports this kinetic limitation. It gives a characteristic diffusion time of 59 s for the path of 42.5 μm between bulk reservoir and membrane center, which is on the same order of magnitude determined for τ_*q*_V_, incr_ and τ_*q*_V_, decr_. Note that the sample contracts faster by a factor of approximately 2 than it is expanding. This can be rationalized by the distinct mesoscopic mechanisms upon contraction and expansion. In particular, upon potential reversal, the highly strained Si lattice supports the expulsion of the ions.

## DISCUSSION

**Fig. 4:**
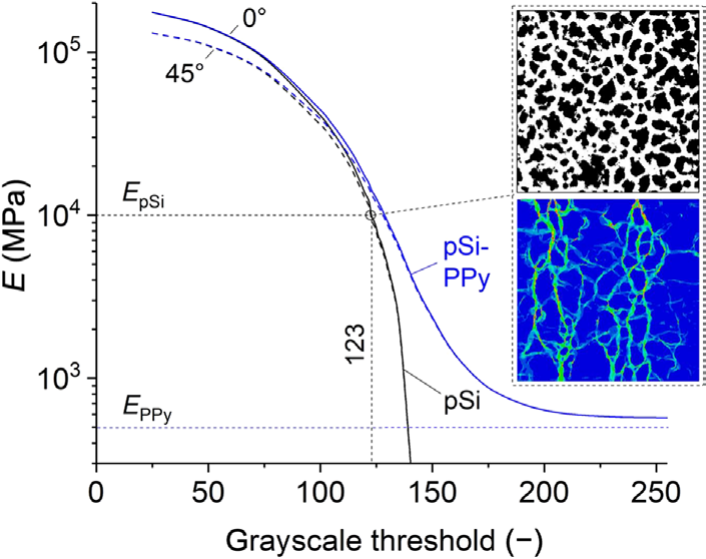
Young’s modulus of the empty and PPy-filled pSi membrane. Values are predicted as function of the grayscale threshold value. The black curve corresponds to the empty pSi membrane, and the blue curve is predicted for the PPy-filled pSi membrane. Calibration of the pSi membrane to the measured macroscopic Young’s modulus of *E* = 10 GPa yields a grayscale threshold of 123.

For obtaining a further understanding of the electroactuation mechanism of the PPy-filled pSi membranes, the micromechanical properties are modeled on the basis of the microstructure as extracted from the electron micrograph of the same area of material shown in Fig. 1A. Details on the translation of the grayscale image into a finite-element model are given in Materials and Methods (*17*–*19*). For the segmentation of the image, a threshold value is used ranging from 0 to 255, discriminating between PPy (dark areas) and Si (bright areas). The predicted curves for the macroscopic Young’s modulus as function of the grayscale threshold are shown in Fig. 4 for the empty and PPy-filled pSi membrane. The results are computed for orientations of the Si <110> crystallographic direction at angles of 0^∘^ and 45^∘^ relative to the *x* axis of the model. For the image with a threshold of 123, the predicted Young’s modulus of the empty pSi membrane under uniaxial tension orthogonal to the pore axis fits the measured macroscopic Young’s modulus *E* = 10 GPa, a value derived from dynamical mechanical analysis (DMA); see sample fabrication. At *E* = 10 GPa (grayscale threshold = 123), the anisotropy of Si is negligible. Hence, the structure of the pSi network dominates the macroscopic stiffness of the material. The top inset in Fig. 4 shows the resulting microstructure of the pSi network (white) and pores (black), being highly irregular. For macroscopic tension in *y* direction, the stress paths through the pSi network appear in green in the von Mises stress distribution in the bottom inset, whereas unstressed areas appear in blue. A comparison of both images reveals that only about 70% of the mass of the pSi network is contributing to the load transfer from the bottom to the top. Along with the progressive drop of the Young’s modulus of pSi in Fig. 4 toward a threshold of 138, this indicates that the network is close to its percolation threshold.

Toward the minimum and maximum grayscale values of 0 and 255, the curves approach the bulk Si and PPy properties, respectively. At the grayscale threshold of 123, calibrated with the macroscopic pSi membranes Young’s modulus, the simulations show a difference in the PPy-filled and empty pSi membrane modulus, which is *E*_pSi − PPy_ − *E*_pSi_ ≈ 4 GPa. The magnification of the effect by a factor of 8 relative to the Young’s modulus of PPy results from the improved connectivity of the pSi network, i.e., the PPy filling acts as a glue that enables a transfer of load through additional Si walls, which do not contribute to load bearing in the empty pSi membrane.

**Fig. 5:**
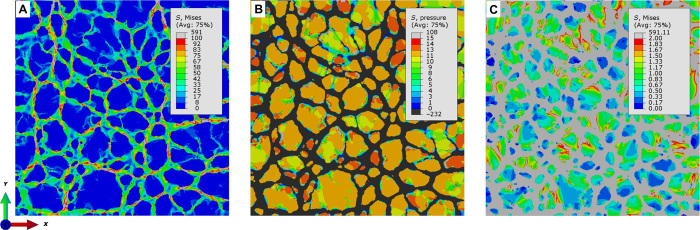
Micromechanical analysis of electrochemical actuation. Results of numerical simulations at maximum swelling strain: (**A**) von Mises stress distribution in the pSi walls, (**B**) pressure distribution in the PPy-infiltrated pores, and (**C**) von Mises stress distribution in the pores—red colored areas exceed the 2-MPa yield stress of PPy.

With the help of a pSi-PPy composite model, the swelling of the PPy in the pores is determined from the macroscopically measured expansion ε = 0.05% of the PPy-filled pSi membrane, as measured from Fig. 2F, yielding the stress-free swelling of the PPy ε_swell, PPy_ = 0.77%. Simulation results for the PPy-filled pSi membrane at maximum swelling strain of the PPy are shown in Fig. 5. Recently, a value of ψ = 0.17 mm^3^/C for the charge-strain coupling parameter of a PPy film that is clamped to a substrate was reported (*13*). The volumetric charge of 0.04 C/mm^3^ that is incorporated in our sample during the linear voltage increase from 0.4 to 0.9 V would thus strain a PPy film by the value of ε = 0.68%. This value is close to our result of ε_swell, PPy_ = 0.77%, although the PPy is not in the shape of a film but incorporated into pSi.

Visible stresses in the pSi network, shown in Fig. 5A as von Mises stress, range from 50 to 100 MPa. In contrast to uniaxial tension, the swelling of the PPy leads to a more isotropic and uniform stress distribution in the Si walls, such that 83% of the wall material is effectively strained. It can be seen from Fig. 5B that smaller pores tend to show higher pressures up to 15 MPa, while larger pores, which may consist of a number of clusters of smaller pores and mechanically inactive Si walls, show pressures in the range from 8 to 12 MPa. Reducing the upper limit of the von Mises stress in the contour plot shown in Fig. 5C to the yield strength of 2 MPa for PPy, as determined by Gnegel *et al.* (*20*), reveals that despite the high pressure in the pores, the polymer reaches the critical stress at which plastic flow occurs. Therefore, the numerical simulations support plasticity as a possible reason for the observed differences in the actuation versus charging kinetics.

In classic all-solid-state piezoelectric systems, a mechanical pressure [“piezo,” derived from the Greek $\pi \iota \overset{\prime }{\varepsilon }\zeta \varepsilon \iota \nu$ (piezein), which means to squeeze or press] couples to a macroscopic electrical polarization and thus to an electrical field via a deformation and thus movements of charged building blocks sitting on a crystalline lattice without inversion symmetry. This is not the case for our system. Here, an internal mechanical swelling pressure in the aqueous electrolyte/PPy system builds up by movements of counter ions into pore space as a function of an electrical potential applied to the entire porous medium with respect to the electrolyte. Thus, a capacitive electrical charging of the entire volume of the porous medium is coupled to a deformation via a charge movement–induced pressure.

How does the electrochemical actuator functionality of our composite compare with state of the art piezoelectric actuation? The piezoelectric coefficient of arguably the best-performing commercially available lead and lead-free piezoelectric ceramics {lead zirconate titanate [PZT; (Pb_0.85_Ba_0.15_)_0.9925_ La_0.005_(Zr_0.52_Ti_0.48_)O_3_] and BZT-50BCT [Ba(Ti_0.8_Zr_0.2_)O_3_ − (Ba_0.7_Ca_0.3_)TiO_3_]}, amounting to *d*_33_ = 410 pCN^−1^ and 620 pCN^−1^ (*21*, *22*), respectively. Thus, to achieve a strain amplitude of ε = 0.05% for the same sample dimensions (*l* = 0.626 mm), as studied here, a potential of 763 or 504 V would have to be applied (see also Fig. 3C). This is 840 and 560 times higher than for our lead-free, biocompatible pSi-PPy material, evidencing the exceptional actuation performance of our hybrid system.

Unfortunately, our step-coulombmetry experiments, discussed above, indicate that the functional kinetics is limited by the diffusion and drift dynamics of the counter ions in pore space. This results in long switching times, on the scale of seconds, much worse than achievable in piezoelectric systems, where switching times on the millisecond scale are easily reached. Here, a hierarchical pore structure encompassing nanopores with large actuation performance connected by macropores with fast bulk-like ion transport, similarly as often established in biological pore or capillary networks to optimize transport, could lead to a substantial increase in the dynamic functional performance (*23*). Given the reversibility of both the counter-ion movement in the nanopores and of the mechanical response of the matrix, the electrochemomechanical coupling should also result in an incorporation or expulsion of ions and thus in an electrical voltage between porous medium and electrolyte, when an external pressure is applied to the porous medium in an open-circuit configuration. This so-called piezoionic effect is still controversially debated in the literature, being attributed to stress gradient induced ion motion, to Donnan potentials arising at the PPy/electrolyte interface, or to the superposition of both (*24*). However, for this reverse, sensoric function, we expect the coupling coefficient to be particularly small, since it should be inversely proportional to the large charge-strain coupling parameter A* determined above. Thus, our composite cannot compete with standard piezoelectric materials in terms of electrical voltage generation. Nevertheless, it will be interesting to explore this possible sensoric material functionality in the future.

A large electrochemical actuation integrated into a mainstream semiconductor, along with the huge varieties of structuring, surface modification, and functional integration of pSi (*7*, *8*), establishes particularly versatile and sustainable pathways to couple in an adaptive manner electronics with electrochemical energy storage (*25*), actuorics (*26*–*28*), fluidics (*29*, *30*), sensorics (*6*, *31*), and photonics (*32*) in aqueous electrolytic media. It thus extends the previous approaches to combining classic piezoelectric actuator materials, e.g., by thin-film on-silicon coatings and epitaxial heterostructures to achieve on-chip actuorics, most prominently in the realm of nano- and micromechanical systems (*26*, *33*). Similarly, as established in nature in many biological composites (*9*), the remarkably good functionality of the material results from a multiscale combination of light elemental constituents, here, H, C, N, O, Si, and Cl. In particular, no heavy metals, most prominently Pb, as omnipresent in high-performance piezoceramics, are necessary for the functionality. Given the biocompatibility and biodegradability of both pSi (*8*) and PPy (*34*), along with the exceptionally small operation voltages, this integrated material system may be particularly suitable for biomedical actuoric functions. From a more general materials science perspective, our study demonstrates how the advent of self-organized porosity in solids in combination with self-assembly and functionalization on the single-pore scale allows one to bridge the gap between bottom-up and top-down approaches for the design of 3D mechanical robust materials, a particular challenge for embedding functional nanocomposites in macroscale devices (*35*).

## MATERIALS AND METHODS

### Sample fabrication

The silicon single crystals are p-doped and have a resistivity of 0.01 to 0.02 ohm ∙ cm, a (100) orientation, and a thickness of 525 μm. One side has been polished by the supplier (Si-Mat silicon Materials GmbH). The wafer is contacted by a thin piece of aluminum foil and mounted into an electrochemical setup with an inner radius of 1.58 cm. It is made of polytetrafluorethylene to safely handle hydrofluoric acid (HF). Initially, the native silicon dioxide layer on the surface of the silicon wafer is stripped by immersing the silicon in a 1% aqueous HF solution for 30 s. The cell is then filled by a volumetric 4 : 6 mixture of HF (48%; Merck EMSURE) and ethanol (absolute; Merck EMSURE). A round platinum counter electrode (CE) is implemented above the silicon in the electrochemical cell so that the platinum is submerged in the electrolyte. A current is applied between the platinum CE (cathode) and the silicon working electrode (anode), to etch pores into the silicon. The applied current amounts to 98 mA, which is equivalent to a current density of 12.5 mA/cm^2^. It is applied for 100 min. In a final step, the current is increased to detach the pSi layer from the remaining bulk silicon by means of an electro polishing process. Therefore, the current is set to 2 A for 30 s. The resulting detached porous layer is rinsed three times with deionized water (18.2 megohm) and left to dry under ambient conditions for 2 hours.

The part of the fabricated pSi membrane, which is intended to be filled by a PPy polymerization, has a size of 1.008 cm^2^. A thin gold layer is deposited onto the detached side of the membrane. It has a thickness of 20 ± 0.5 nm. As already described in the results section, the membrane is placed in an electrochemical cell, and a platinum pseudo–reference electrode (RE) and a platinum CE are inserted into the cell (see fig. S1B). The platinum RE has a stable potential of 0.2 V versus an Ag/AgCl RE. The solution for the electropolymerization is then added, which contains 0.1 M pyrrole monomers (Sigma-Aldrich) and 0.1 M lithium perchlorate (LiClO_4_) (Sigma-Aldrich) in acetonitrile (LiChrosolv, Merck) as a solvent. The pyrrole monomer is distilled at 130^∘^C under ambient conditions immediately before the polymerization process to obtain a pyrrole solution with a high monomer amount. The LiClO_4_ is dissolved by stirring for 5 min. The solution is transferred to the electrochemical cell and allowed to imbibe into the pores for 10 min. The constant current of 0.403 mA, which is applied for the electropolymerization, corresponds to a current density of 400 μA/cm^2^ per sample area. The reference voltage is recorded in steps of 0.1 s during the process. Afterward, the sample is rinsed three times with deionized water and allowed to dry under ambient conditions for 2 hours.

The charge *C* consumed in the electropolymerization can be related to the weight *W*_theor._ of the polymerized pyrrole by

$$W_{\text{theor}.}=\frac{C\cdot M}{z\cdot F}$$ (1)

where the Faraday constant *F* equals 96485.332 C/mol. *M* gives the formula weight of PPy, obtained from the molar mass of a monomer, while including incorporated ${\text{ClO}}_{4}^{-}$ counter ions and amounts to 96.78 g/mol (*36*). *z* is the number of electrons used in the process to polymerize two monomers and is estimated by Diaz *et al.* (*37*) to 2.25. For the electropolymerization shown in Fig. 1B, *C* amounts to 7.605 C, which results in a theoretical weight of *W*_theor._ = 0.00339 g. The weight of the whole sample increases from 0.01005 g, measured before the polymerization, to 0.01350 g afterward. Hence, the actual weight increase is *W*_meas._ = 0.00345 ± 0.00001 g and coincides well with the theoretical expectation.

Scanning electron microscopy (SEM) micrographs with EDX are recorded to verify whether the PPy is distributed equally along the whole thickness of the pSi membrane. The nitrogen signal in the EDX data is homogenous over the sample profile, which suggests a homogenous filling of the membrane over its thickness *t*. SEM micrographs are recorded with a Zeiss Supra 55 VP electron microscope at a potential of 5 kV. TEM micrographs are recorded with a FEI Talos F200X.

The sample is electrically contacted at the bottom by connecting a gold wire to the deposited gold layer. Subsequently, the sample is mounted. Therefore, the bottom of the sample is encased in epoxy for approximately 1 cm. The sample is then placed in a glass beaker and installed in the dilatometer. It can then be immersed in HClO_4_. The sample that is in contact with the acid has a volume of *V*_sample_ = 0.186 mm^3^ and the volume of the pore space, using the porosity Φ, amounts to *V*_pores_ = 0.093 mm^3^.

The electrochemical measurements are conducted in 1 M perchloric acid. The acid is prepared from 70% HClO_4_ (Suprapur, Merck) and deionized water. The measurements are performed with a potentiostat equipped with a linear scan generator (Metrohm Autolab PGSTAT 30) in a three-electrode setup. A reversible hydrogen electrode (Gaskatel HydroFlex) serves as the RE and a carbon fabric as the CE.

The dilatometry measurements are conducted with a Linseis L75 dilatometer. Initially, the quartz pushrod of the dilatometer is carefully lowered onto the membrane with a static force of 0.4 N to install the sample into the dilatometer. It is effectively able to detect changes in the membrane length *l* with a resolution of 12.5 nm.

The measurements to determine the macroscopic Young’s modulus of an empty pSi membrane are performed in a DMA (Netzsch DMA 242 C) with a three-point bending setup. Thereby, a pSi membrane with a thickness of 100 μm is positioned on two contacts on the side, while a pushrod applies a sinusoidal force in the middle of the sample until a certain strain amplitude is reached. From the force and the strain, a Young’s modulus can be derived. The Young’s modulus of 10 GPa presented in this work has been found by averaging over many load and unload cycles and upending the pSi membrane and repeating the measurements.

### Micromechanical analysis

The 2D finite element (FE) method model of the pSi-PPy membrane is set up with the same discretization as the TEM, which is a .tiff image consisting of 512 × 512 pixels. Assuming that the microstructure is constant through the thickness of the pSi membrane, each pixel of the image is represented by a four-node quadrilateral plain strain element CPE4 in ABAQUS (*38*). The properties of each element are chosen according to the corresponding grayscale value of the image. For values below the grayscale threshold, the element is part of the pore space, and for values equal or above the threshold, the element is added to the element set representing the Si network. The resulting model consists of 262,144 equally sized quadrilateral four-node elements organized in two element sets for assigning the Si and pore material properties. Orthotropic elasticity is used to model the pSi membrane in the frame of a reference of a standard (100) silicon wafer, which is [110], [110], and [001] with *E*_1_ = *E*_2_ = 169 GPa and *E*_3_ = 130 GPa; ν_23_ = 0.36, ν_31_ = 0.28, and ν_12_ = 0.064; and *G*_23_ = *G*_32_ = 79.6 GPa and *G*_12_ = 50.9 GPa (*39*). Simulations are carried out for two orientations with [110] in *x* direction of the model (0^∘^) and [110] oriented 45^∘^ relative to the *x* direction of the model (45^∘^). For this rotation, the *z* axis and three-direction [001] serve as common rotation axis for the Si crystal. For the simulation of the pure pSi membrane, the element set for the pores is assigned with isotropic elasticity *E*_p_ = 1 MPa and ν_p_ = 0.35, i.e., the pore space has a negligible but nonzero stiffness. With this, all elements in the model are properly connected, and the simulation is numerically stable. For simulations with a PPy filling inside the pore space, the elasticity parameters of the pore elements are set to *E*_p_ = *E*_PPy_ = 500 MPa and ν_p_ = ν_PPy_ = 0.35 (*13*). Swelling of PPy is implemented by assigning a thermal expansion of α_PPy_ = 1 K^−1^ such that ε_swell, PPy_ = ε_th, PPy_ = αΔ*T*, where temperature difference Δ*T* serves as an adjustable parameter. To model the effect of swelling as the sole effect of PPy, the thermal expansion for Si is set to α_Si_ = 0 K^−1^. For all simulations in ABAQUS, the boundaries are forced to remain plane using *EQUATION. Displacement boundary conditions are applied to the bottom and left boundaries such that the nodes do not move normal to the boundaries. The displacements of the top and right boundaries are controlled via dummy nodes included in the *EQUATION constraints. For macroscopic tension, the dummy node at the top boundary is displaced in positive *y* direction. The *x* displacement of the dummy node at the right boundary can be used to determine the Poisson’s ratio. For the swelling simulation, the displacements of both dummy nodes at the top and right boundaries are left free to move normal to the respective boundary.

Further processing and analysis of the binarized image are performed in several steps in the open-source program Fiji (*40*). As the skeletonization is very sensitive to surface irregularities, the image is smoothed and again binarized. The smoothing process replaces each pixel with the average of its 3 × 3 neighborhood. During this process, the volume fraction is increased by negligible 0.001. The skeleton is constructed with the surface-thinning algorithm by Lee *et al.* (*41*), which is implemented as the process Skeletonize3D in the plugin BoneJ (*42*) in Fiji. It converts an image object into its one-voxel-wide inner skeleton centerline. If no surface smoothing is performed before this, then the resulting skeleton consists of 2633 branches compared to 584 and 1461 junctions compared to 342.

For the thickness measurement of the pSi walls, the Quasi-Euclidean 2D option of the Distance Transformation in the MorphoLibJ plugin (*43*) is used. The resulting Euclidean distance transform (EDT) image is multiplied with the skeleton image. As the resulting EDT values correspond to half of the wall thickness, the EDT values along the skeleton are multiplied by two. The same process was introduced by Badwe *et al.* (*44*) and Stuckner *et al.* (*45*). Besides the average wall thickness, the nonlocal values can be analyzed, as described in Richert and Huber (*19*).

The average pore size of the smoothed and binarized image is computed using the thickness calculation (*46*) in the plugin BoneJ (*42*). It constructs the diameter information at each voxel with the Biggest Sphere algorithm by Hildebrand *et al.* (*47*). As this algorithm attributes one value to each pore space voxel, the average is volume-based instead of skeleton-based as the EDT approach, which makes it more suitable for the pore space.

Grosman *et al.* (*48*) analyzed the macroscopic Young’s modulus of pSi, assuming that the Si walls are organized in a honeycomb structure, using the in-plane elastic constants from (*49*). From the discrepancy of the measurements with the calculated values, the authors concluded that the Young’s modulus of the Si walls is five times smaller than that of bulk silicon and explained this with the finite-size effect that can decrease the effective Young’s modulus.

However, because of the strong irregularity of the network and the uncertainty concerning the threshold, it is not trivial to characterize the geometry of the pSi network. Here, the calibration of the grayscale threshold via the macroscopic Young’s modulus provides a very sensitive measure for the segmentation of the image into Si and pore space, which can then be analyzed using Fiji (*40*). The segmentation determines the fraction of Si in the membrane to 0.508, which is in excellent agreement with the measured porosity Φ = 50%. Using the Euler distance transformation (*43*) and image multiplication with the skeleton (*41*, *42*), the average thickness of the Si walls is determined to *t*_Si_ = 9.95 nm ≈ 10 nm, with an SD of 4 nm. These values are used in the following for a comparison with the predicted properties based on a honeycomb structure.

For completeness, the average diameter and SD of the pores is measured with the volume-based Biggest Sphere algorithm (*47*), yielding 18.9 and 6. nm, respectively. The junctions between walls are on average threefold connected, which equals the connectivity in a honeycomb structure. The average wall length in the fully interconnected network is determined as 19.1 nm. Besides the average values, the network can furthermore be analyzed nonlocally, which is here achieved by object-oriented programming, as in the work of Richert and Huber (*19*). The average thickness of a wall along its axis is not constant but can be classified as a slightly concave profile. At the junctions, the network is on average 13.0 nm thick and, at the thinnest part of a wall, on average 8.6 nm thick.

For an average wall size of *t* = 10 nm and a porosity of 50%, the computed wall length of a honeycomb structure is *l* = 20 nm. According to (*17*), the effective Young’s modulus under uniaxial tension is given by

$$E=\frac{4}{\sqrt{3}}{\left(\frac{t}{l}\right)}^{3}E_{\mathrm{Si}}$$ (2)

Inserting *t*/*l* = 0.5 and *E* = 10 GPa yields an estimate for *E*_Si_ of only 35 GPa, which is comparable to the value reported in (*48*). However, our analysis shows that 30% of the Si material carries little or no load (see inset in Fig. 4). Furthermore, considering the measured SD of the wall thickness, which is almost 50% of the average wall thickness, the idealization with a regular honeycomb structure is highly questionable. For instance, reducing the *t*/*l* ratio in Eq. 2 to *t*/*l* = 0.31 already yields a Young’s modulus of *E*_Si_ = 145 GPa, which corresponds to the average bulk Young’s modulus of Si. Therefore, the high sensitivity of the macroscopic stiffness with respect to the *t*/*l* ratio along with the drop in structural stiffness caused by partially deactivated Si walls [see also (*18*) for 3D networks], underlines the importance of a careful characterization and translation of the pSi membrane into a micromechanical model that reflects the real irregular microstructure, as shown in the top inset of Fig. 4.

## Supplementary Material

aba1483_SM.pdf

## SUPPLEMENTARY MATERIALS

Supplementary material for this article is available at http://advances.sciencemag.org/cgi/content/full/6/40/eaba1483/DC1
