# Supplementary material for: Giant electrochemical actuation in a nanoporous silicon-polypyrrole hybrid material
Source: Sci Adv. 2020 Sep 30;6(40):eaba1483. doi: 10.1126/sciadv.aba1483 (PMC7527211; doi:10.1126/sciadv.aba1483)
Supplement: aba1483_SM.pdf [file aba1483_SM.pdf]

Science Advances
AAAS

advances.sciencemag.org/cgi/content/full/6/40/eaba1483/DC1

Supplementary Materials for

Giant electrochemical actuation in a nanoporous silicon-polypyrrole hybrid material

Manuel Brinker, Guido Dittrich, Claudia Richert, Pirmin Lakner, Tobias Krekeler, Thomas F. Keller, Norbert Huber, Patrick Huber*

*Corresponding author. Email: patrick.huber@tuhh.de

Published 30 September 2020, *Sci. Adv.* **6**, eaba1483 (2020)
DOI: 10.1126/sciadv.aba1483

This PDF file includes:

Fig. S1

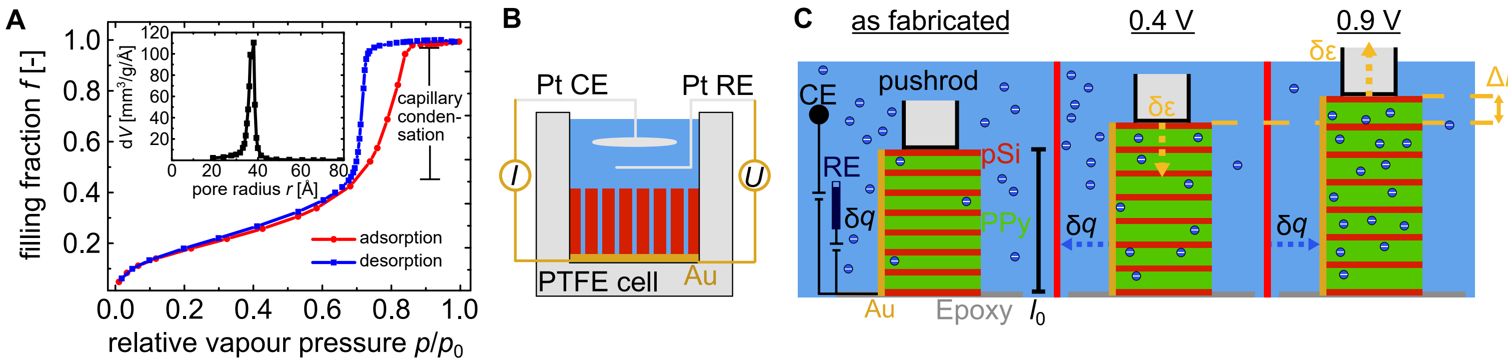

Fig. S1. Structural characterization by sorption isotherm, illustration of the polymerization cell and illustration of the electroactuation setup. (**A**) Nitrogen sorption isotherm at $T = 77\,\mathrm{K}$ recorded for nanoporous silicon. Plotted is the volume filling fraction f against the relative vapour pressure p/p_0. The inset depicts the resulting pore radius r distribution. (**B**) Electrochemical cell for the polymerization of PPy in the pSi membrane. The membrane is contacted via a gold layer. The current is applied via a platinum counter electrode (CE) while the voltage is measured by a platinum wire acting as a pseudo reference electrode (RE). (**C**) Schematics of the electroactuation experiments. The pSi membrane filled with PPy is immersed in 1M perchloric acid ($\mathrm{HClO_4}$) and contacted via the gold layer. The current is applied between a carbon counter electrode (CE) while the voltage is measured by a standard hydrogen reference electrode (RE). The right part symbolizes the case where a voltage of $0.9\,\mathrm{V}$ is applied and the anions are incorporated into the PPy resulting in the expansion of the sample. Vice versa, in the middle part a voltage of $0.4\,\mathrm{V}$ is applied and the anions are expelled followed by the subsequent contraction of the sample.
